# Neurotensin protects pancreatic beta cells from serum deprivation

**DOI:** 10.1080/13813450802536034

**Published:** 2009-01-05

**Authors:** Eleni Dicou

**Affiliations:** ^a^ Department of Biochemistry and Molecular Biology, University of Texas Medical Branch, Galveston, Texas 77555-0652, USA

In a recent article entitled “Neurotensin protects pancreatic beta cells from apoptosis” Coppola *et al*. (2008) show that neurotensin (NT) protects insulin-producing cells (*β*-TC3, INS-1E) against IL-1*β* and staurosporine (STS)-induced cell death.

NT responses are known to be mediated through three receptors. Two NT receptors (NTSR1 and NTSR2) belong to the family of G-protein coupled receptors (GPCRs). The third NT receptor, NTSR3, also called sortilin, belongs to the family of the receptors related to the yeast sorting receptor Vps10p. All three receptors are expressed in these cells, although NTSR2 expressed in *β*-TC3 has a different molecular weight (MW) from that in INS-1E cells, which in turn has a different MW from that expected for NTSR2. According to the authors “discrepancies are the consequences of distinct phospholipids environments leading to different solubilities in detergent”. However, they provide no references to support this assumption and, in addition, specificity controls, i.e. pre-adsorption of the serum by its immunogenic peptide, that would strengthen this point, were not done.

The NTSR1 antagonist SR48692 did not reverse the protective action of NT on IL-1*β* induced cell death while the NTSR2 agonist, levocabastine, had the same protective action as NT, thus implicating NTSR2 in this protection. NT in IL-1*β* treated cells reduced caspase-3 activity. Levocabastine had the same effect as NT on caspase-3, while SR48692 had no effect; the NTSR3 propeptide released from the precursor form of NTSR3, and described as an antagonist of NT, also had no effect (Munck Petersen *et al*., 1999). Paradoxically, equimolar amounts of NTSR3 propeptide were used in this study, while at least 10-fold higher concentrations are needed to effectively antagonize NT, thus no conclusion can be drawn as to its NT-antagonizing/caspase-3-activating effect.

No data is given as to the percent of *β*-TC3 and INS-1E cell death induced by STS, as determined by Hoechst 33342 staining. Some effect of NT on the STS-induced caspase-3 activity was seen after 8 hours, but the effect was not followed even for 24 hours.

In Figure 5 (of Coppola *et al*., 2008) some indirect evidence is given suggesting that the PI-3 kinase pathway is involved in the NT effect against STS-induced cell death (individual effects of the kinase inhibitors are not shown). Unfortunately the study provides little information on the mechanisms involved in the NT protection against IL-1*β* induced cell death, and it would have been more probing that the authors explored further this effect instead of studying also the STS-induced effect. Cytokine-induced cell death of pancreatic beta cells implicated nitric oxide (NO) and prostaglandin E2 production, due to the up-regulation of the inducible form of NO synthase and cyclo-oxygenase-2, JAK/STAT activation and NF-*κ*B activation, and it would be of interest to evaluate whether NT interferes with any of these pathways (Eizirik *et al*., 2008).

For unexplained reasons, IL-1*β* and STS are added in cell cultures in serum-free medium. However, in all recent articles IL-1*β* and STS are added to pancreatic cell lines in culture in serum-supplemented medium (Ortis *et al*., 2006; Tejedo *et al*., 2004). It is well known that *β*-TC3 and INS-1E are clonal insulin-producing cells that depend on serum for their survival. So, the reported 2 to 5% cell death for the control cells seems low for these conditions.

**Figure 1.**
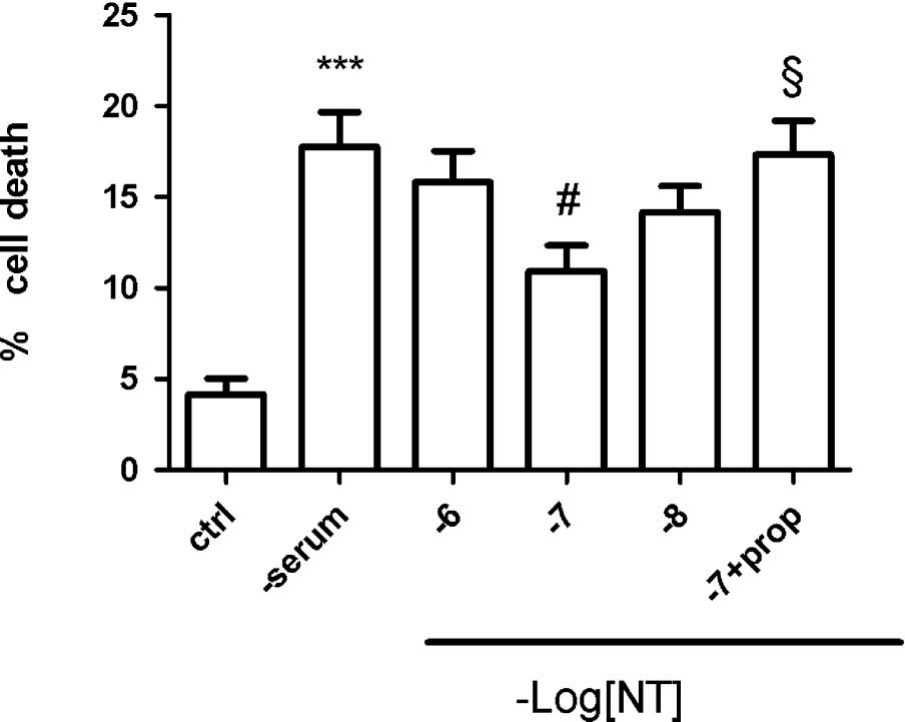
Neurotensin protects *β*-TC3 pancreatic cells against serum deprivation-induced cell death. Cells were cultured in serum-free medium for 48 hours. NT was added at the concentrations indicated at 0 hour and 24 hours of starvation. The NTSR3 propeptide (prop) was added at 1 *μ*M. Controls were in presence of serum. After 48 hours viable cells were determined using the Hoechst 33342 dye. Means ± SEM are from five independent experiments. ***p < 0.001 as compared to control; ^#,§^ *p* < 0.05 as compared to – serum and 10^−7^ M NT respectively, by Student's *t*-test.

As shown in the Figure 1, there is a very significant cell death of *β*-TC3 cells grown to about 70% confluency in RPMI-1640, 10% FCS, 2 mM glutamine, penicillin (100 U/ml) streptomycin (100 μg/ml) and followed by a 48 hour serum deprivation, as measured with the Hoechst 33342 dye. Addition of 0.1 *μ*M NT significantly protected against cell death and this effect was also significantly reversed by the addition of 1 *μ*M NTSR3 propeptide, thus implicating sortilin in mediating the NT protective effect against cell death by serum deprivation. Additional experiments should be performed in primary beta cells to clarify the role of NT for beta cell survival.

Sortilin together with p75NTR was shown to mediate neuronal cell death by proneurotrophins and excess of NT reversed this effect (Nykjaer *et al*., 2004; Teng *et al*., 2005). However, the pathway involved in sortilin mediated cell death is not yet known. Several evidences link cell death due to serum deprivation to a pathway independent of caspase activation, via mitochondrial pro-apoptotic factors such as AIF (Joza *et al*., 2001; Pandey *et al*., 2003), while cytokine-induced and STS-induced apoptosis in pancreatic cells are caspase dependent.

In summary, different death stimuli in pancreatic cells may involve different NT receptors and be mediated via different pathways.
